# Supplementary material for: Light-modulated stem cells in the camera-type eye of an annelid model for adult brain plasticity
Source: Nat Commun. 2025 Dec 1;16:9861. doi: 10.1038/s41467-025-65631-0 (PMC12669781; doi:10.1038/s41467-025-65631-0)
Supplement: Supplementary file 1 — Supplementary Information [file 41467_2025_65631_MOESM1_ESM.pdf]

Supplementary Information

Light-modulated stem cells in the camera-type eye of an annelid model for adult brain plasticity

Nadja Milivojev, Federico Scaramuzza, Pedro Ozório Brum, Camila L. Velastegui Gamboa, Gabriele Andreatta, Florian Raible, Kristin Tessmar-Raible

Supplementary information includes:

Supplementary Figures 1-11
Supplementary Reference

Additional materials for this manuscript are deposited at the Zenodo archive:

https://doi.org/10.5281/zenodo.17349847

containing:

- FACS Plots 1-4
- Genome Mapping 1-4
- R code
- R objects

Raw scRNA-seq data have been deposited at the European Nucleotide Archive (ENA) under the European Nucleotide Archive under the project accession code (project accession PRJEB98620, samples SAMEA120303337 to SAMEA120303348), accessible at the following URL: https://www.ebi.ac.uk/ena/browser/view/PRJEB98620.

This contains the following samples:

- Biosample SAMEA120303337 – 175739 – immature
- Biosample SAMEA120303338 – 175740 – premature
- Biosample SAMEA120303339 – 175741 - female
- Biosample SAMEA120303340 – 175742 - male
- Biosample SAMEA120303342 – 205588 - EdU-positive cells
- Biosample SAMEA120303341 – 205584 - sampling time-matched EdU control
- Biosample SAMEA120303343 – 287885 - *copsin1*$^{\Delta 8/\Delta 8}$_1
- Biosample SAMEA120303344 – 287886 - *copsin1*$^{\Delta 8/\Delta 8}$_2
- Biosample SAMEA120303345 – 287887 - *copsin1*$^{\Delta 8/\Delta 8}$_3
- Biosample SAMEA120303346 – 287888 - *copsin1*$^{+/+}$_1
- Biosample SAMEA120303347 – 287889 - *copsin1*$^{+/+}$_2
- Biosample SAMEA120303348 – 287890 - *copsin1*$^{+/+}$_3

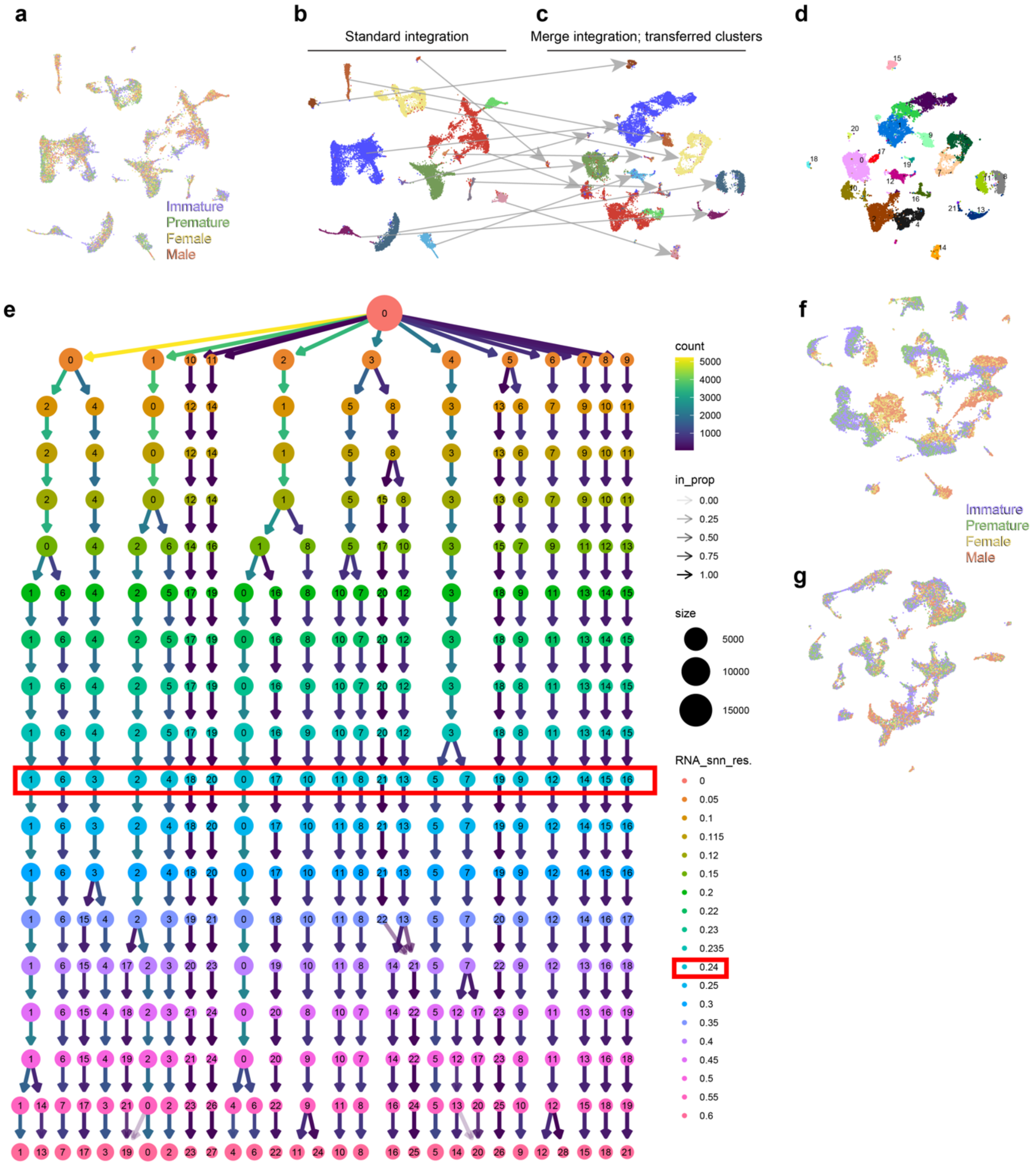

Supplementary Figure 1. Robustness of the head/brain atlas to clustering methodology and sample size. (**a-d**) Overall equivalence between clusters generated by standard integration and merge integration pipelines: (a,b) UMAP representation of a map produced by integration of immature, premature, female and male libraries following the standard pipeline of the R package Seurat; (c,d) analogous map produced by merge-integration, as also presented in **Fig. 1b-e** of this study; matching colors and arrows in (b,c) link cluster identities matched between the different representations; **(e)** Clustering tree representing increasing clustering resolutions (RNA_snn_res) and the resulting cluster numbers; box highlights the chosen level of granularity; **(f,g)** UMAP representations of clustering results for data without down-sampling of libraries; using (f) merge-integration and (g) standard integration pipelines, respectively.

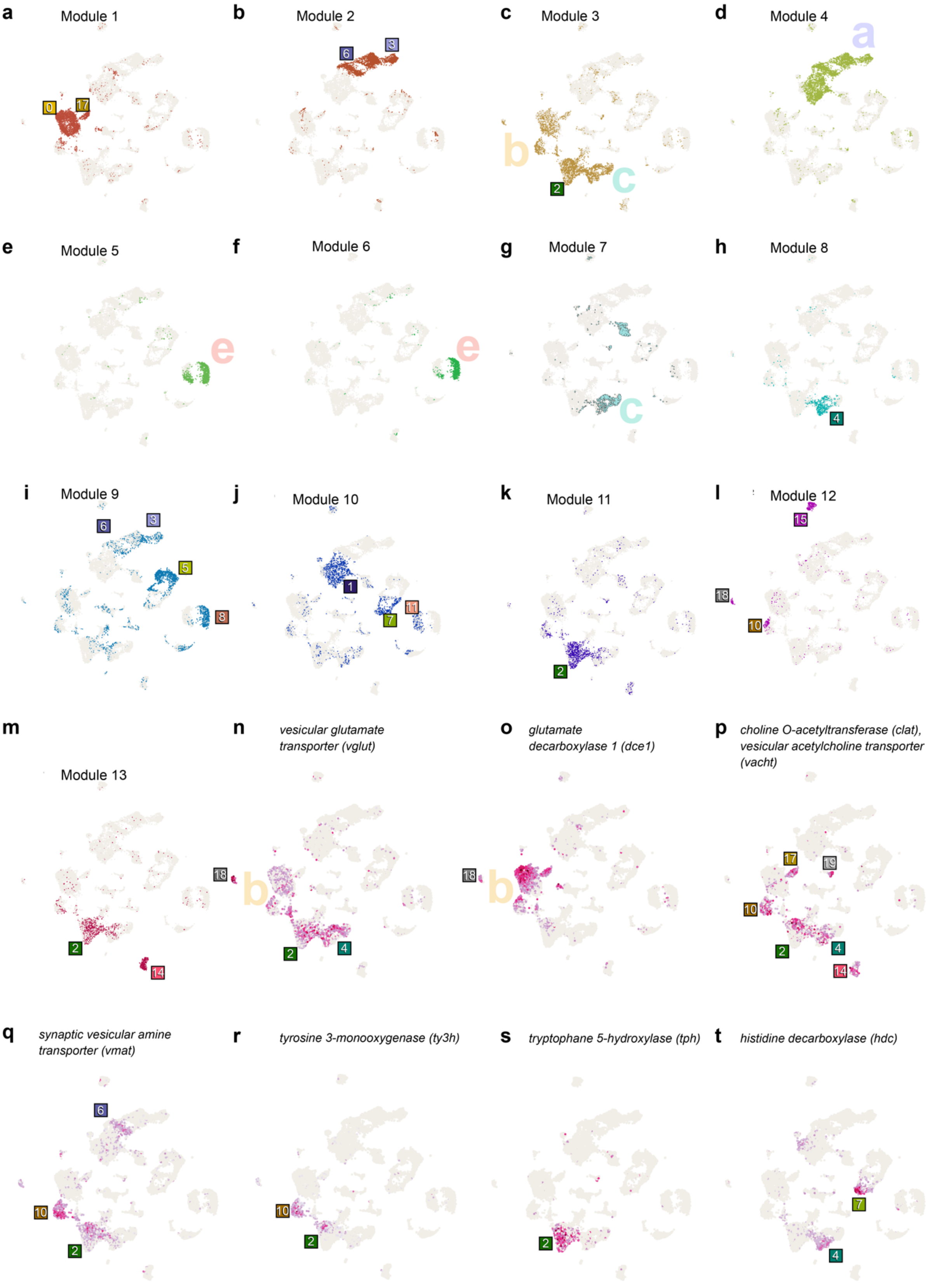

Supplementary Figure 2. Gene co-expression modules and diagnostic markers for different neurotransmitter systems support the division into cell populations and super-clusters as biologically relevant groupings. (**a-m**) Identified gene co-expression modules projected onto the UMAP representation of the head/brain atlas; **(n-t)** visualisation of the expression of markers of different neurotransmitter systems. Also see Supplementary Data 2 and 5.

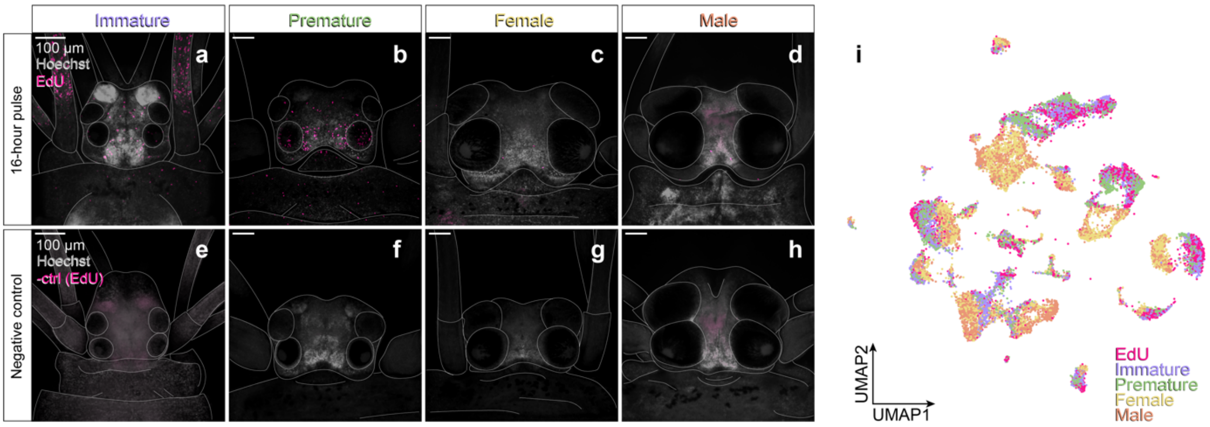

Supplementary Figure 3. EdU-positive cells in the *Platynereis* head and representation of EdU-positive cell signatures on the head cell atlas.
(**a-h**) Non-inverted images and respective controls for the 16h EdU incorporation assays shown in **Fig. 2a-d**; (**i**) EdU-positive cell transcriptomes projected on the UMAP representation of the merge-integrated head cell atlas dataset (also see **Fig. 2f** for visualisation of a gene module produced from EdU-library-enriched genes).

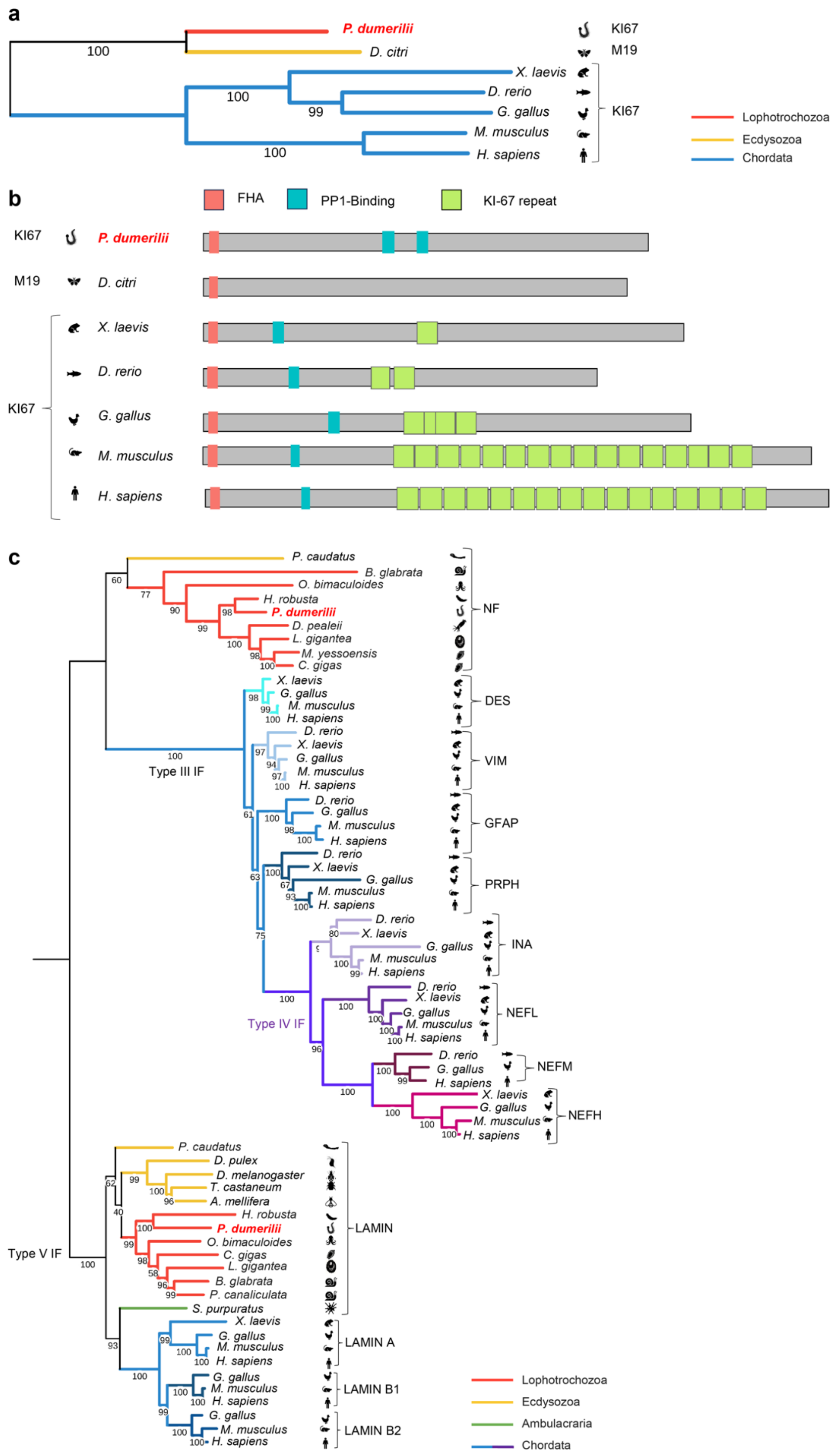

Supplementary Figure 4. Phylogeny of *bona fide* neurogenesis- and proliferation- associated genes. (**a**) Phylogeny of Ki67; unrooted tree. (**b**) Schematic representation of protein domains corresponding to proteins shown in (a). **(c)** Phylogeny placing *Platynereis* Nf as an invertebrate Neurofilament/Nf protein, joint orthologs of mammalian GFAP and other filament proteins. Lamin proteins were used as an outgroup. Bootstrapping percentages shown in internal branches. See Supplementary Data 5 for protein identifiers.

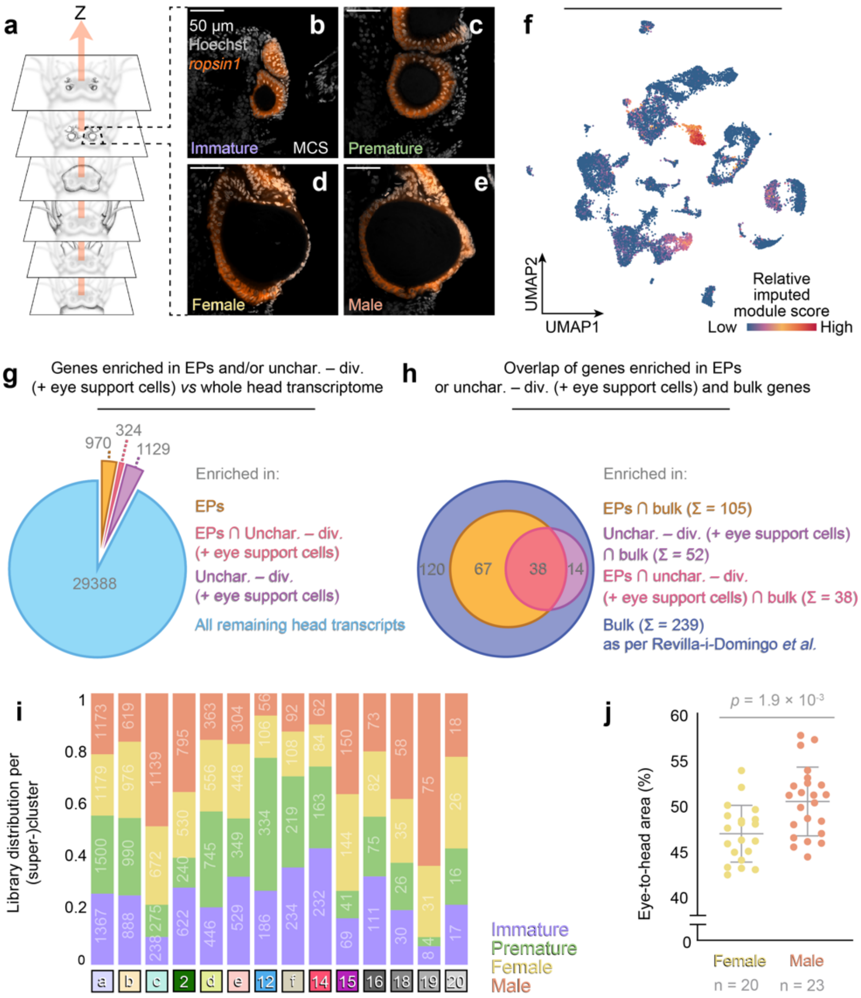

Supplementary Figure 5. ***In situ*** **and** ***in silico*** **identification of eye growth.**
(**a**) Schematic representation showing the placement of the focal planes imaged in (b-e). (**b-e**) *In situ* HCR staining of *r-opsin1* (red) in (**b**) immature, (**c**) premature, (**d**) female and (**e**) male eye. Microscopy images displayed as 1 µm thick medial cross-sections of the posterior eye. (**f**) Visualisation on the head/brain map of a cumulative gene module, based on genes either enriched exclusively in eye photoreceptors or jointly enriched in eye photoreceptors and trunk *r-opsin1*-expressing cells, as detected by bulk RNA-sequencing in ref. 1; in further text referred to as "bulk". Relative Venn diagrams of (**g**) genes enriched in EPs and uncharacterised – diverse (+ eye support cells) against the whole head transcriptome, as well as their overlap, and (**h**) the overlap of genes exclusively or jointly enriched in EPs and/or uncharacterised – diverse (+ eye support cells) and bulk genes. (**i**) Representation of immature, premature, female and male scRNAseq libraries in clusters and super-clusters of the brain atlas; numbers inside the bars signify individual cell numbers. (**j**) Comparison of eye size of female (n = 20) and male (n = 23) worms. Mean values ± SD are displayed as cumulative area of four eyes per animal normalised to the head area. Statistics: unpaired t-test.

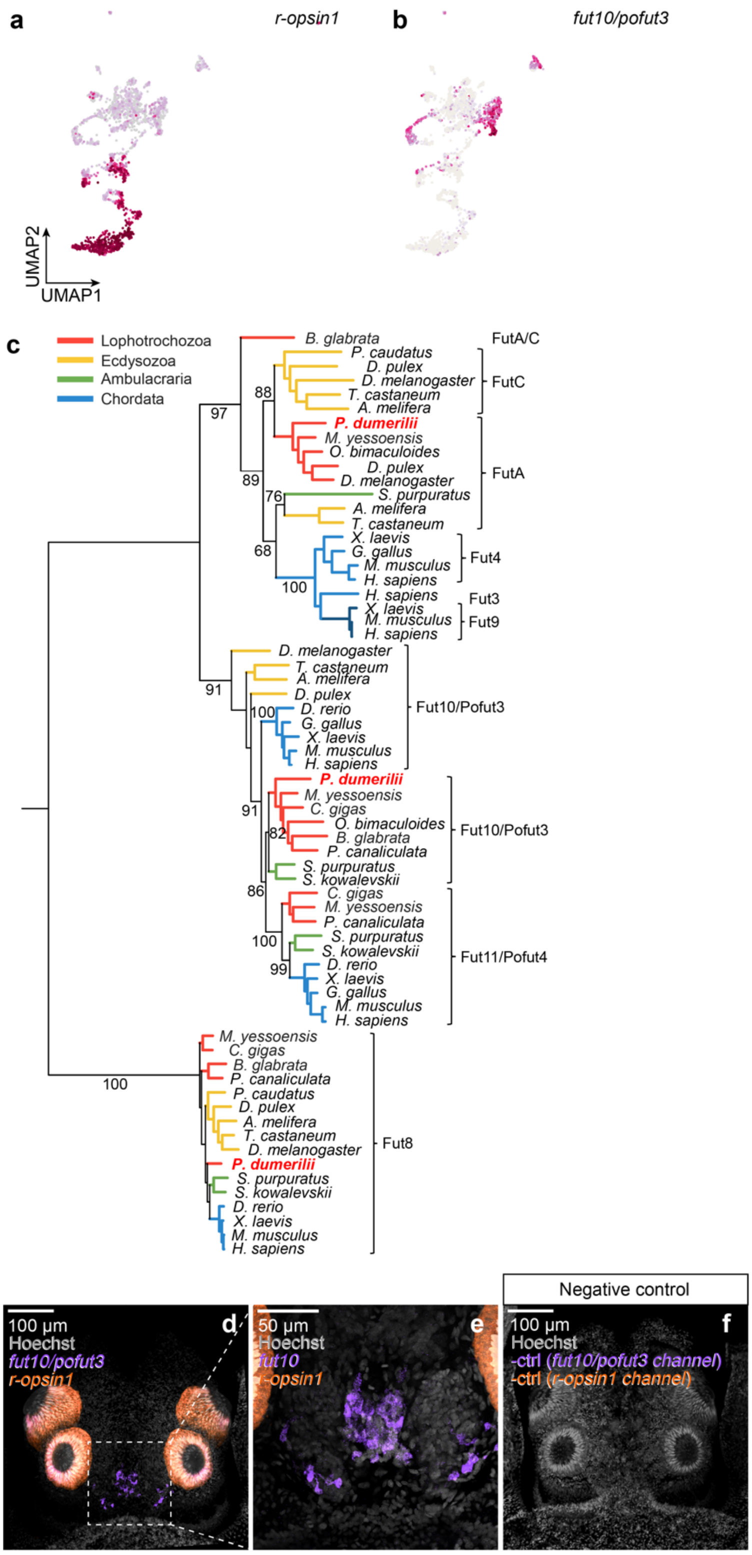

Supplementary Figure 6. Phylogeny and expression of *fut10/pofut3*.
(a,b) Visualisation in the eye- and transcriptomically related cell subset of (a) the EP marker *r-opsin1* and (b) the tentative eye support-cell marker *fut10/pofut3*. (**c**) Phylogenetic tree placing *Platynereis* Fut10/Pofut3 into the larger family of Fut proteins; bootstrapping percentage shown in internal branches. Tree was rooted using Fut8 as an outgroup. (**d-f**) HCR detection of *fut10/pofut3* expression in the head and corresponding negative control. Microscopy images displayed as dorsoventral stacks of the head, of 51, 25 and 37 µm respective thickness. Sequence identifiers for phylogenetic analyses: see Supplementary Data 5. HCR probes: Supplementary Data 11.

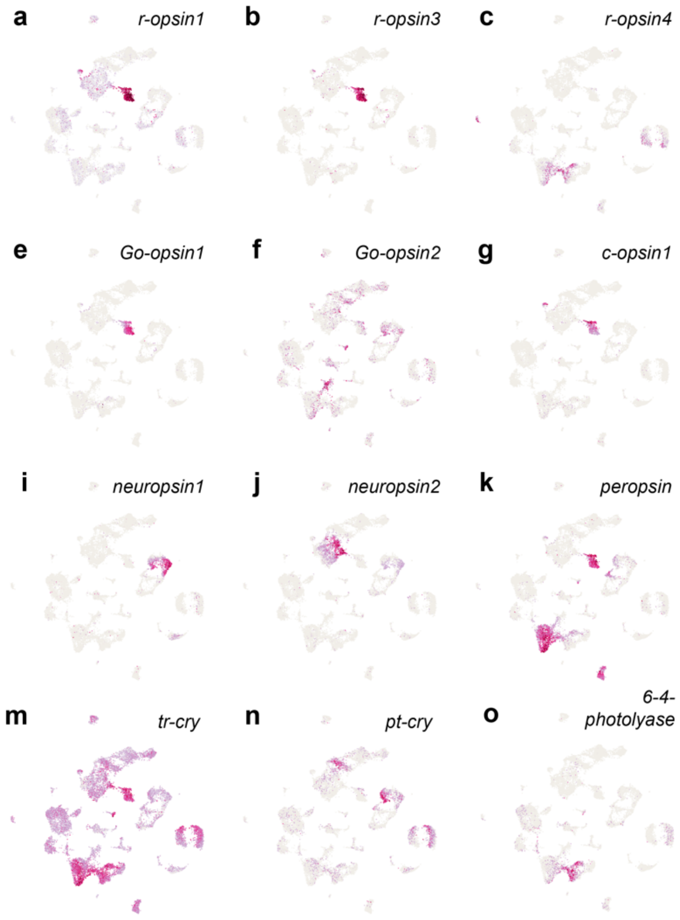

Supplementary Figure 7. Expression of known genes for light-sensitive proteins in the *Platynereis* head/brain atlas.
(**a-o**) Transcript expression predicted by scRNA-seq of known light-sensitive proteins in the *Platynereis* head/brain atlas. The survey omits genes for some light-sensitive proteins: *r-opsin2* lacked sufficient reads, and for *CPD-photolyase*, the gene model in the current reference genome was incomplete. See Supplementary Data 5 for gene identifiers.

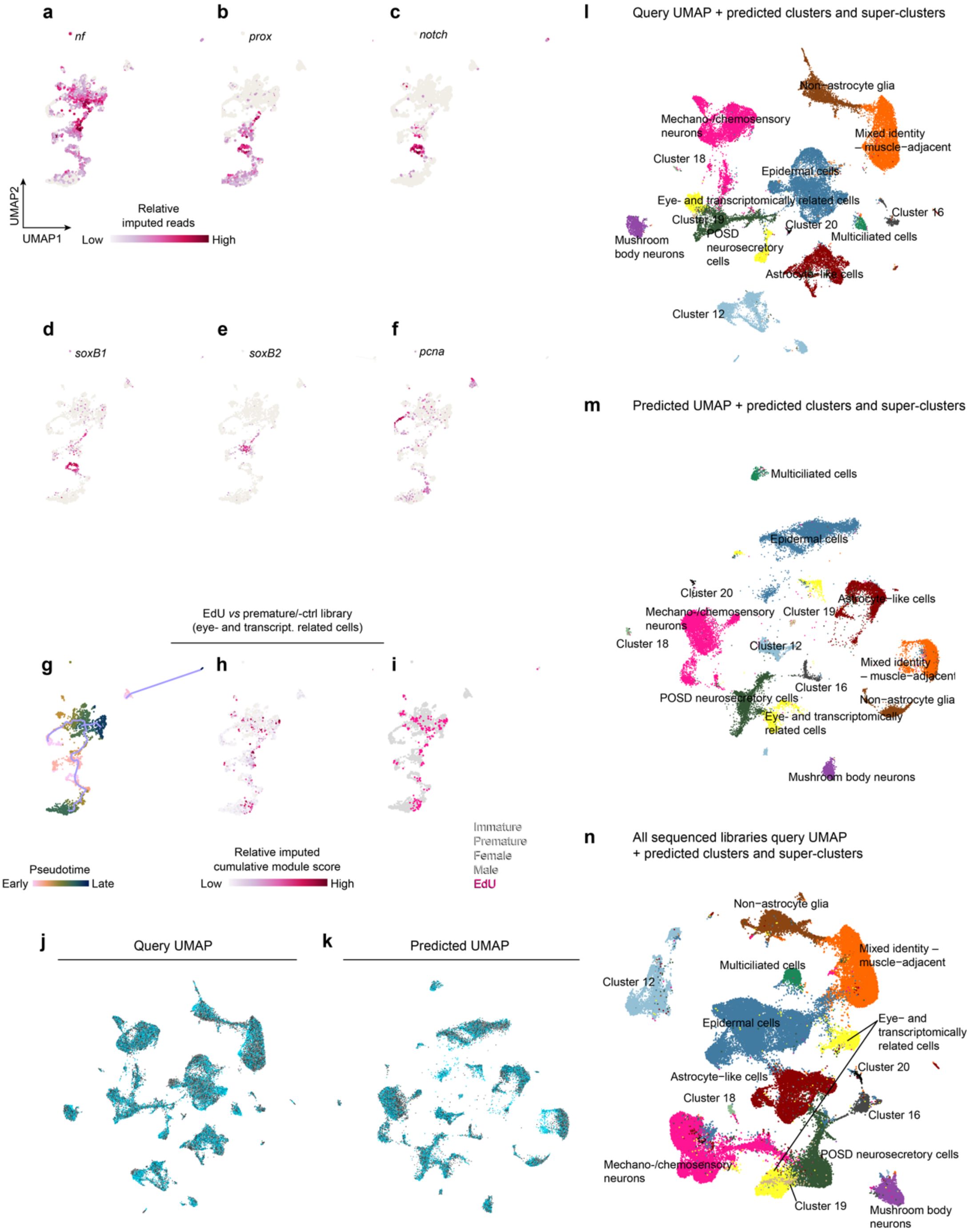

Supplementary Figure 8. Inferred differentiation trajectories of eye stem cells, and stability of cluster identities in datasets including the additional *c-ops1*$^{\Delta8/\Delta8}$ and WT scRNAseq data.
(a-f) Visualisation of (a) *nf,* (b) *prox*, (c) *notch*, (d) *soxB1*, (e) *soxB2* and (f) *pcna* in the eye- and transcriptomically related cell subset; (**g**) Tentative differentiation and pseudotime ranking of cells in the dataset. (**h,i**) Representation of the EdU-sorted library in the sub-clustered dataset, by (h) visualisation of a cumulative module computed from the eye- and transcriptomically related cell subset of the EdU library and the premature and sampling time-matched control libraries, respectively; (i) Spatial distribution of the EdU library transcriptomes in the dataset. Consistent with the extended, 96-hour pulse, most labelled cells locate to cell populations inferred to be lineage-committed or differentiated cells. (**j, k**) UMAP representations of the *c-ops1*$^{\Delta8/\Delta8}$ and WT dataset; (j) standard dimensional reduction of the dataset ("Query UMAP"); (k) projection on the head/brain atlas UMAP reference by predicting the UMAP-coordinates of the query using the coordinates of the reference ("Predicted UMAP"). (**l, m**) Predicted super-cluster identities visualised for (l) the "Query UMAP"-reduction in panel (j), or (m) the predicted UMAP-reduction in panel (k). (**n**) Predicted clusters and super-clusters visualised on a "Query UMAP"-reduction of a dataset consisting of all libraries generated in the scope of the study (69153 cells).

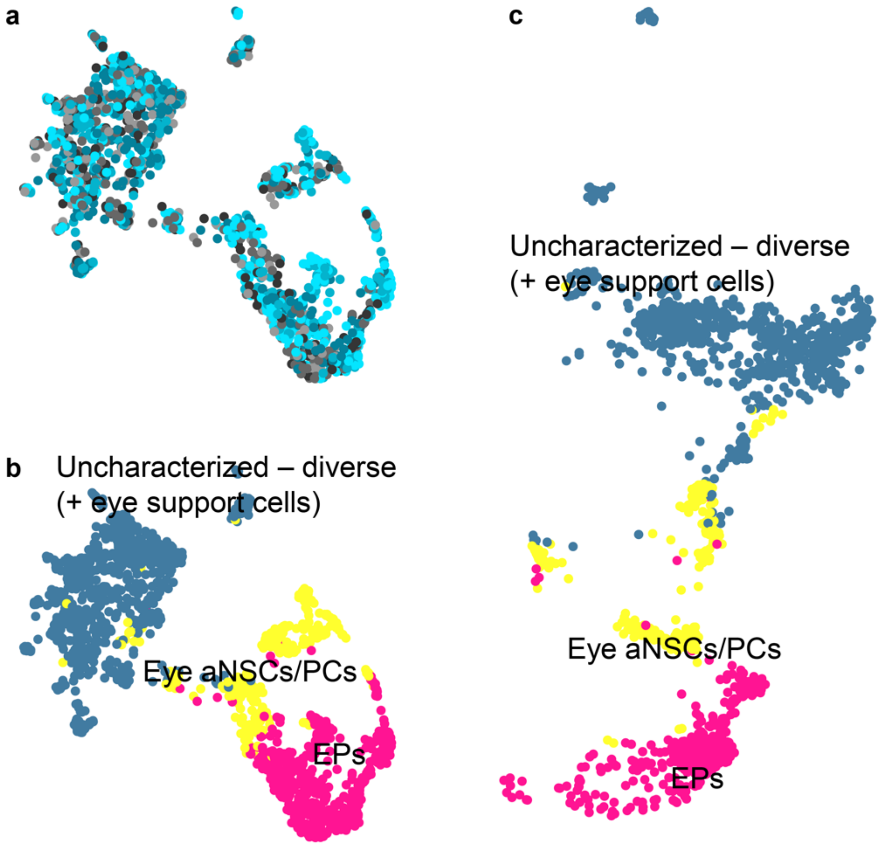

Supplementary Figure 9. Predicted reduction and annotation of the eye subset of the *c-ops1*$^{\Delta 8/\Delta 8}$ and wild-type dataset.
(**a**) Standard UMAP-reduction of the *c-ops1*$^{\Delta 8/\Delta 8}$ and WT eye- and transcriptomically-related cell subset (query). Colour code as in **Fig. 5**. **(b, c)** Predicted partitions visualised in (b) the query UMAP-reduction and, respectively, (c) the predicted UMAP-reduction of the eye- and transcriptomically-related cell subset, generated using the UMAP-coordinates and partitions of the corresponding subset of the head/brain atlas as a reference.

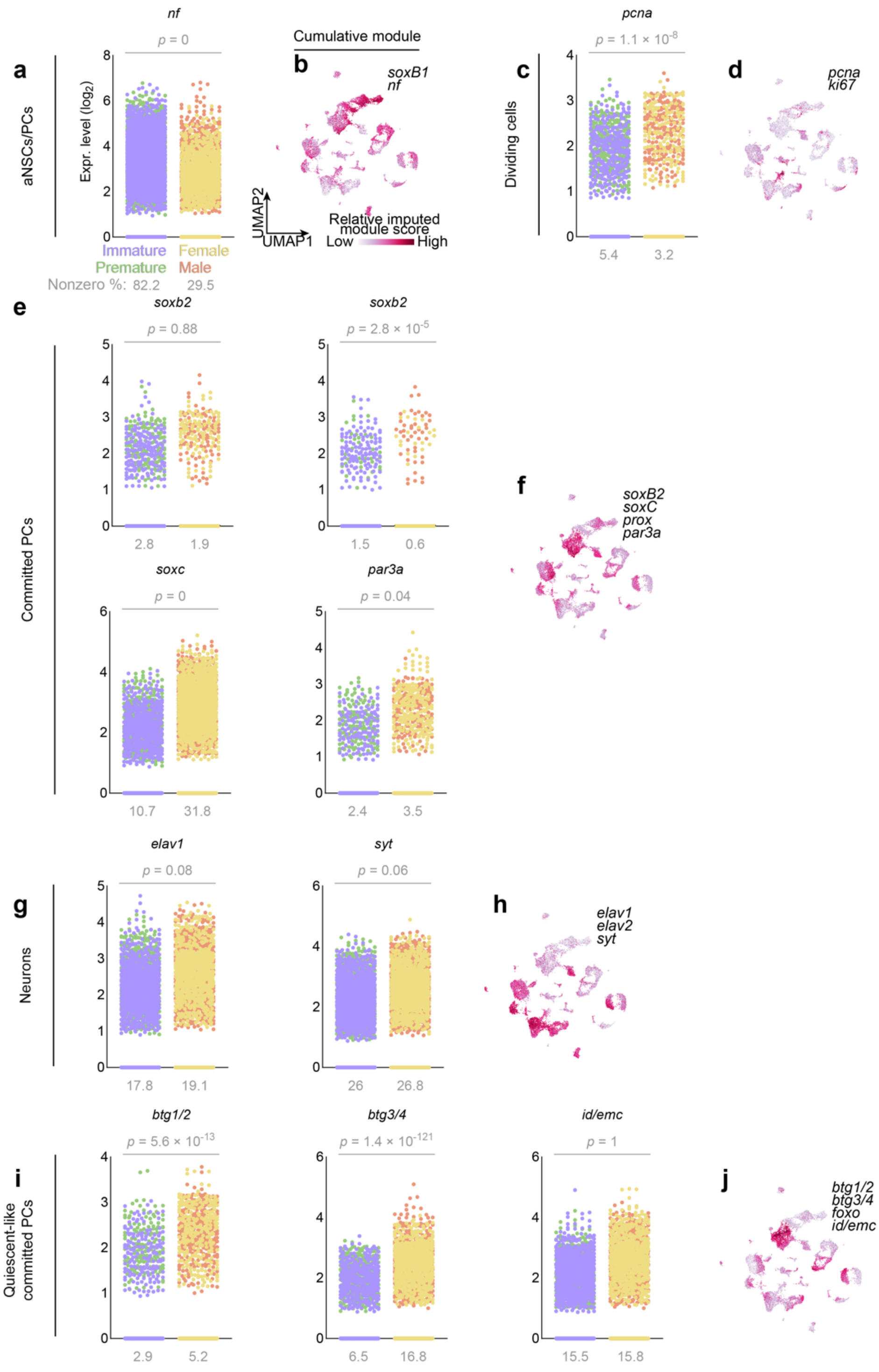

Supplementary Figure 10. Combined upregulation of proliferation- and quiescence-like signatures in mature animals. Scatterplots and cumulative gene modules showing respective individual and cumulative expression of genes tentatively associated with (**a,b**) aNSCs/PCs, (**c,d**) proliferation, (**e,f**) neural commitment, (**g,h**) post-mitotic neurons and (**i,j**) quiescence. Statistical significance of expression means of non-reproductive and reproductive libraries was calculated using a Wilcoxon Rank Sum test. Markers are inferred from mammalian signatures, and not strictly limited to neuronal populations. For additional scatterplots, cf. **Figure 6g-k**. Separate plots for *soxb2* in (e) reflect a split locus in the available reference genome annotation (see Supplementary Data 5).

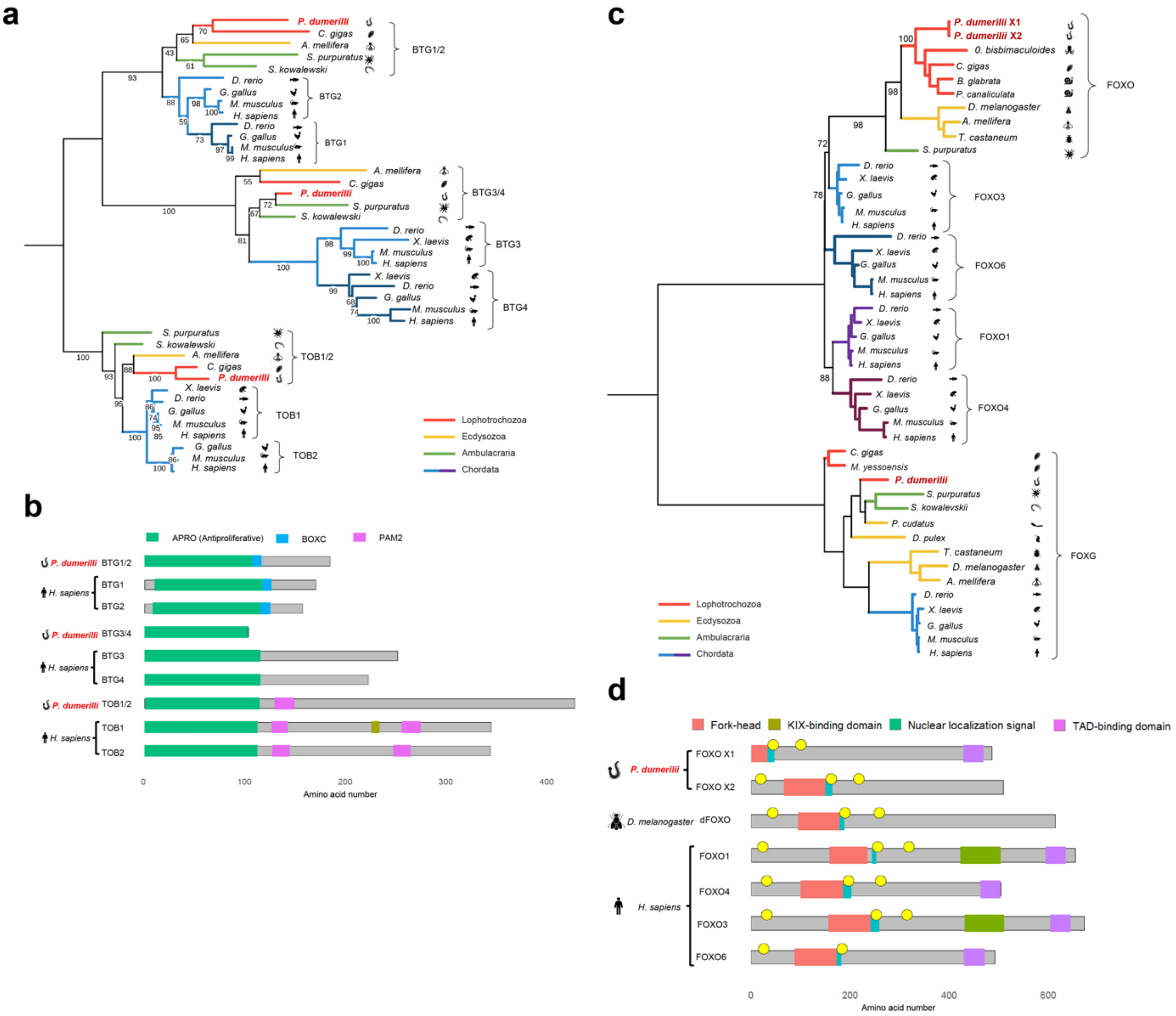

Supplementary Figure 11:Phylogeny of newly identified *Platynereis* orthologs of mammalian quiescence-associated genes.

(**a**) Phylogenetic tree of the BTG/TOB protein family; statistical support by bootstrapping percentage shown in internal branches. Tree was rooted TOB family as an outgroup. (**b**) Schematic representation of protein domains corresponding to proteins shown in (a) in *Platynereis* and *H. sapiens*. (**c**) Phylogenetic tree of FoxO proteins; statistical support by bootstrapping percentage shown in internal branches. Tree was rooted with FoxG members as outgroup. (**d**) Schematic representation of protein domains corresponding to proteins shown in (C) in Platynereis, *D. melanogaster* and *H. sapiens*. Sequence identifiers are provided in Supplementary Data 5.

Supplementary Reference

1. Revilla-i-Domingo, R. *et al.* Characterization of cephalic and non-cephalic sensory cell types provides insight into joint photo- and mechanoreceptor evolution. *Elife* **10**, e66144 (2021).
