# Supplementary material for: Light-modulated stem cells in the camera-type eye of an annelid model for adult brain plasticity
Source: Nat Commun. 2025 Dec 1;16:9861. doi: 10.1038/s41467-025-65631-0 (PMC12669781; doi:10.1038/s41467-025-65631-0)
Supplement: Supplementary file 3 — Description of Additional Supplementary Files [file 41467_2025_65631_MOESM3_ESM.pdf]

Description of additional supplementary material

• **Supplementary Data 1:**
Head / Brain atlas cluster markers. Libraries from immature, premature, female and male worms were integrated into a single dataset (brain atlas); markers of resulting cells clusters are listed; statistics based on two-sided Wilcoxon Rank Sum tests with Bonferroni correction.

• **Supplementary Data 2:**
Head / Brain atlas gene correlation modules. Independent gene correlation analysis was conducted to identify modules of co-expressed genes and cell populations enriched in the modules.

• **Supplementary Data 3:**
Quantification of eye sizes and EdU-positive cells. Eye growth in worms of different reproductive stages, as well as c-ops1Δ8/Δ8 and corresponding wild-type (WT) worms was quantified through eye size measurements, as well as comparison of the number of proliferating (EdU-labeled) cells.

• **Supplementary Data 4:**
Differential expression between entire libraries from EdU-labeled and unlabeled cells (premature library of the brain atlas, as well as a premature control library sampled alongside the EdU library), as well as their respective eye-and transcriptomically related cell subsets; statistics based on two-sided Wilcoxon Rank Sum tests with Bonferroni correction.

• **Supplementary Data 5:**
Identifiers of proteins and genes used in this study.

• **Supplementary Data 6:**
Genes enriched in eye photoreceptors and uncharacterized – diverse (+ eye support cells) and overlap with genes exclusively enriched in eye photoreceptors and jointly enriched in eye photoreceptors and trunk r-opsin1 expressing cells as per Revilla-i-Domingo et al. (referred to as "bulk"). Statistics: two-sided negative binomial distribution test.

• **Supplementary Data 7:**
Partition markers of the eye- and transcriptomically related cells subset. Clusters of the eye- and transcriptomically related cells subset were grouped manually into partitions based on marker genes and their in situ expression; markers of the manually assigned identities are presented; statistics based on two-sided Wilcoxon Rank Sum tests with Bonferroni correction.

• **Supplementary Data 8:**
Differential expression analysis between non-reproductive and reproductive libraries. Differential expression was performed between whole grouped immature and premature (non-reproductive) and female and male (reproductive) libraries; statistics based on two-sided Wilcoxon Rank Sum tests with Bonferroni correction.

• **Supplementary Data 9:**
GO term analysis non-reproductive vs reproductive libraries. GO terms of genes differentially expressed in libraries of non-reproductive specimens are presented; terms pertaining to proliferation, neurogenesis, as well as hallmarks of quiescence, senescence and cell death were copied from the AmiGO 2 database and individually looked up.

• **Supplementary Data 10:**
GO term analysis reproductive vs non-reproductive libraries. Analogous data to Supplementary Data 9, but for genes differentially expressed in libraries of specimens at reproductive stages.

• **Supplementary Data 11:**
Sequences of HCR probes used in the study to investigate spatial expression patterns of selected genes produced by the analyses.

• **Supplementary Movie 1:**
3D rendering of a posterior eye after 16 h EdU labeling (specimen 1). EdU-positive cells (yellow) are primarily localized in the vicinity of the eye opening, at the edge of the cup-shaped retina. Nuclei are counterstained with DAPI (gray). The apparent flattening of the eye cup is due to mounting of specimens for microscopy.

• **Supplementary Movie 2:**
3D rendering of a posterior eye after 16 h EdU labeling (specimen 2). EdU-positive cells (yellow) are primarily localized in the vicinity of the eye opening, at the edge of the cup-shaped retina. Nuclei are counterstained with DAPI (gray). The apparent flattening of the eye cup is due to mounting of specimens for microscopy.

• **Supplementary Movie 3:**
3D rendering of a posterior eye after 16 h EdU labeling (specimen 3). EdU-positive cells (yellow) are primarily localized in the vicinity of the eye opening, at the edge of the cup-shaped retina. Nuclei are counterstained with DAPI (gray). The apparent flattening of the eye cup is due to mounting of specimens for microscopy.
