# Supplementary material for: Light-modulated stem cells in the camera-type eye of an annelid model for adult brain plasticity
Source: Nat Commun. 2025 Dec 1;16:9861. doi: 10.1038/s41467-025-65631-0 (PMC12669781; doi:10.1038/s41467-025-65631-0)
Supplement: Supplementary file 18 — Reporting Summary [file 41467_2025_65631_MOESM18_ESM.pdf]

nature portfolio

Corresponding author(s): Florian Raible, Kristin Tessmar-Raible

Last updated by author(s): Sep 18, 2025

Reporting Summary

Nature Portfolio wishes to improve the reproducibility of the work that we publish. This form provides structure for consistency and transparency in reporting. For further information on Nature Portfolio policies, see our Editorial Policies and the Editorial Policy Checklist.

Statistics

For all statistical analyses, confirm that the following items are present in the figure legend, table legend, main text, or Methods section.

n/a	Confirmed	
☐	☒	The exact sample size (*n*) for each experimental group/condition, given as a discrete number and unit of measurement
☐	☒	A statement on whether measurements were taken from distinct samples or whether the same sample was measured repeatedly
☐	☒	The statistical test(s) used AND whether they are one- or two-sided<br>*Only common tests should be described solely by name; describe more complex techniques in the Methods section.*
☒	☐	A description of all covariates tested
☐	☒	A description of any assumptions or corrections, such as tests of normality and adjustment for multiple comparisons
☐	☒	A full description of the statistical parameters including central tendency (e.g. means) or other basic estimates (e.g. regression coefficient) AND variation (e.g. standard deviation) or associated estimates of uncertainty (e.g. confidence intervals)
☐	☒	For null hypothesis testing, the test statistic (e.g. *F*, *t*, *r*) with confidence intervals, effect sizes, degrees of freedom and *P* value noted<br>*Give P values as exact values whenever suitable.*
☒	☐	For Bayesian analysis, information on the choice of priors and Markov chain Monte Carlo settings
☒	☐	For hierarchical and complex designs, identification of the appropriate level for tests and full reporting of outcomes
☒	☐	Estimates of effect sizes (e.g. Cohen's *d*, Pearson's *r*), indicating how they were calculated

Our web collection on statistics for biologists contains articles on many of the points above.

Software and code

Policy information about availability of computer code

Data collection	ZEN Blue 3.3, FACSChorus 3.0, FACSDiva 9.0.1
Data analysis	Cutadapt 1.12, fastQC 0.19, multiQC 1.14, STAR aligner 2.7.10b, featurecounts 2.0.1, CellRanger 7.0.1/8.00, R 4.4.1, RStudio 2023.09, Seurat 4.4.0, Patchwork 1.2.0, Tidyverse 2.0.0, Paleteer 1.6.0, Clustree 0.5.0, Scales 1.3.0, ggsci 3.2.0, dplyr 1.1.4, SeuratWrappers 0.20, scCustomize 1.1.3, fcoex 1.10.0,, Monocle3 1.3.7, BLASTX, BLASTN, BLASTP, IQ-Tree 1.6.12, ShinyGO 0.80, CLC Mainworkbench 22, iTOL Suite, HCR3.0 Probe Maker 2021_0.3.2, GraphPad Prism 10.1.0, Microsoft Excel 2019, Adobe Photoshop 2024, Adobe Illustrator 2023

For manuscripts utilizing custom algorithms or software that are central to the research but not yet described in published literature, software must be made available to editors and reviewers. We strongly encourage code deposition in a community repository (e.g. GitHub). See the Nature Portfolio guidelines for submitting code & software for further information.

Data

Policy information about availability of data

All manuscripts must include a data availability statement. This statement should provide the following information, where applicable:

- Accession codes, unique identifiers, or web links for publicly available datasets
- A description of any restrictions on data availability
- For clinical datasets or third party data, please ensure that the statement adheres to our policy

All data are available in the main text, the supplementary material, and deposited at the Dryad repositories (containing links to additional Zenodo repositories): https://datadryad.org/stash/share/x6pSympEtsaa7bUUflE4s3T1gxDZpy41JK3zxsTjtSU

https://datadryad.org/stash/share/yY-xBsG0_eFFiJDZfSOV3DveKgldQzGESrvintGVouc

The manuscript does not contain clinical or third party data.

Research involving human participants, their data, or biological material

Policy information about studies with human participants or human data. See also policy information about sex, gender (identity/presentation), and sexual orientation and race, ethnicity and racism.

Reporting on sex and gender	N/A
Reporting on race, ethnicity, or other socially relevant groupings	N/A
Population characteristics	N/A
Recruitment	N/A
Ethics oversight	N/A

Note that full information on the approval of the study protocol must also be provided in the manuscript.

Field-specific reporting

Please select the one below that is the best fit for your research. If you are not sure, read the appropriate sections before making your selection.

- [x] Life sciences
- [ ] Behavioural & social sciences
- [ ] Ecological, evolutionary & environmental sciences

For a reference copy of the document with all sections, see nature.com/documents/nr-reporting-summary-flat.pdf

Life sciences study design

All studies must disclose on these points even when the disclosure is negative.

Sample size	scRNA-seq experiments were performed on 20 immature, 10 premature, 8 female and 8 male untreated wildtype worms in order to be able to extract the same amount of cells from each sample. Female and male worms are notably larger than immature worms and slightly larger than premature worms. To extract the same amount of EdU-labeled cells from EdU-treated premature worms, 120 worms were sacrificed. For the scRNA-seq experiment involving wildtype and c-opsin1 mutant worms, 3 replicate samples were sequenced per genotype, with 10 premature worms per replicate. For experiments involving quantification of eye size in female and male worms, 20 and 23 female and male worms were used, respectively. For the quantification of EdU-labeled cells, in worm heads and eyes, 5-8 worms were used per sample to maintain feasibility of manual cell counting and adhere to standard sample sizes in the field.
Data exclusions	No samples were excluded from the study; during the process of filtering scRNA-seq libraries, cells bioinformatically deemed empty or suspected as multiplets were excluded; for unbiased comparison of cell population sizes, libraries in each experiment were normalized to the size of the library with the lowest number of cells.
Replication	All attempts at replication were successful.
Randomization	For each sample/replicate, animals from the same genotype and strain were selected, preferably from sibling batches; if not possible due to the number of available animals, closely related batches were sampled.
Blinding	Manual quantification and phenotype comparison were conducted blindly.

Reporting for specific materials, systems and methods

We require information from authors about some types of materials, experimental systems and methods used in many studies. Here, indicate whether each material, system or method listed is relevant to your study. If you are not sure if a list item applies to your research, read the appropriate section before selecting a response.

Materials & experimental systems

n/a	Involved in the study
☐	☒ Antibodies
☒	☐ Eukaryotic cell lines
☒	☐ Palaeontology and archaeology
☐	☒ Animals and other organisms
☒	☐ Clinical data
☒	☐ Dual use research of concern
☒	☐ Plants

Methods

n/a	Involved in the study
☒	☐ ChIP-seq
☐	☒ Flow cytometry
☒	☐ MRI-based neuroimaging

Antibodies

Antibodies used	Anti-Green Fluorescent Protein Antibody, polyclonal, Aves Labs (GFP-1020)<br>Goat anti-Chicken IgY (H+L) Secondary Antibody, Alexa Fluor 488 coupled, Thermofisher (A-11039)
Validation	Antibody had previously been validated in the species:<br>B. Backfisch,V.B. Veedin Rajan,R.M. Fischer,C. Lohs,E. Arboleda,K. Tessmar-Raible,& F. Raible, Stable transgenesis in the marine annelid Platynereis dumerilii sheds new light on photoreceptor evolution, Proc. Natl. Acad. Sci. U.S.A. 110 (1) 193-198, https://doi.org/10.1073/pnas.1209657109 (2013).

Animals and other research organisms

Policy information about studies involving animals; ARRIVE guidelines recommended for reporting animal research, and Sex and Gender in Research

Laboratory animals	All Platynereis dumerilii worms used in the study were derived from inbred laboratory strains: Vienna PINK wild-type strain; transgenic r-opsin::egfpvbc2 (Backfisch et al.PNAS 2013); c-ops1Δ8/Δ8 mutant (Veedin Rajan et al. Nat Ecol Evol 2021). As animal age was an essential variable in experimental design, sampling criteria are described in detail in the main text, as well as materials and methods of the study. Briefly, immature (asexual), premature (about to undergo reproductive metamorphosis) and reproductive (female and male) worms were sampled based on birth date, body segment count, body morphology, coloration and presence or absence of gametes.
Wild animals	N/A
Reporting on sex	Sex was considered in reproductive animals; both sexes were represented in the study.
Field-collected samples	N/A
Ethics oversight	Experiments on animals were conducted following the applicable Austrian and European guidelines for animal research. No ethical approval was required, as all experiments were performed on invertebrate animals.

Note that full information on the approval of the study protocol must also be provided in the manuscript.

Plants

Seed stocks	*Report on the source of all seed stocks or other plant material used. If applicable, state the seed stock centre and catalogue number. If plant specimens were collected from the field, describe the collection location, date and sampling procedures.*
Novel plant genotypes	*Describe the methods by which all novel plant genotypes were produced. This includes those generated by transgenic approaches, gene editing, chemical/radiation-based mutagenesis and hybridization. For transgenic lines, describe the transformation method, the number of independent lines analyzed and the generation upon which experiments were performed. For gene-edited lines, describe the editor used, the endogenous sequence targeted for editing, the targeting guide RNA sequence (if applicable) and how the editor was applied.*
Authentication	*Describe any authentication procedures for each seed stock used or novel genotype generated. Describe any experiments used to assess the effect of a mutation and, where applicable, how potential secondary effects (e.g. second site T-DNA insertions, mosiacism, off-target gene editing) were examined.*

Flow Cytometry

Plots

Confirm that:

- [x] The axis labels state the marker and fluorochrome used (e.g. CD4-FITC).
- [x] The axis scales are clearly visible. Include numbers along axes only for bottom left plot of group (a 'group' is an analysis of identical markers).
- [x] All plots are contour plots with outliers or pseudocolor plots.
- [x] A numerical value for number of cells or percentage (with statistics) is provided.

Methodology

Sample preparation	Cell suspensions were obtained from dissociated and fixed worm head tissue that was filtered and suspended in a saline solution.
Instrument	BD FACSMelody Cell Sorter/BD FACSAria III Cell Sorter
Software	FACSChorus 3.0/FACSDiva 9.0.1
Cell population abundance	15000 cells were sorted for immature, premature, female, male and EdU-treated samples, comprising ~20% of total events in the former four and 0.68% in the latter. 20000 cells were sorted for the c-opsin1 mutant and corresponding wildtype samples, making up ~13% of all events.
Gating strategy	Primary gate was set to isolate singlets, based on high FSC-H and low-to-medium FSC-A; secondary gate was set to isolate Hoechst-stained cells in G1 and G2, gating populations at 50 and 100 DAPI-A, respectively. The gate for EdU-labeled cells in G1 and G2 was set so that it encompassed a highly mCherry-positive population (fluorophore used: Alexa Fluor 555) that was absent from the negative control.

- [x] Tick this box to confirm that a figure exemplifying the gating strategy is provided in the Supplementary Information.
